# Cardiac Implantable Electronic Devices Infection Assessment, Diagnosis and Management: A Review of the Literature

**DOI:** 10.3390/jcm11195898

**Published:** 2022-10-06

**Authors:** Filippo Toriello, Massimo Saviano, Andrea Faggiano, Domitilla Gentile, Giovanni Provenzale, Alberto Vincenzo Pollina, Elisa Gherbesi, Lucia Barbieri, Stefano Carugo

**Affiliations:** 1 Department of Clinical Sciences and Community Health, University of Milan, 20122 Milan, Italy; 2 Department of Internal Medicine, Fondazione IRCCS Ca’ Granda—Ospedale Maggiore Policlinico, 20122 Milan, Italy

**Keywords:** cardiac implantable electronic device infection, pocket infection, antimicrobial therapy, risk factors, preventive strategies

## Abstract

The use of increasingly complex cardiac implantable electronic devices (CIEDs) has increased exponentially in recent years. One of the most serious complications in terms of mortality, morbidity and financial burden is represented by infections involving these devices. They may affect only the generator pocket or be generalised with lead-related endocarditis. Modifiable and non-modifiable risk factors have been identified and they can be associated with patient or procedure characteristics or with the type of CIED. Pocket and systemic infections require a precise evaluation and a specialised treatment which in most cases involves the removal of all the components of the device and a personalised antimicrobial therapy. CIED retention is usually limited to cases where infection is unlikely or is limited to the skin incision site. Optimal re-implantation timing depends on the type of infection and on the results of microbiological tests. Preventive strategies, in the end, include antibiotic prophylaxis before CIED implantation, the possibility to use antibacterial envelopes and the prevention of hematomas. The aim of this review is to investigate the pathogenesis, stratification, diagnostic tools and management of CIED infections.

## 1. Introduction

Since the first pacemaker (PM) was implanted by Åke Senning in 1958, cardiac implantable electronic devices (CIEDs) have spread worldwide. More sophisticated systems such as implantable cardioverter defibrillators (ICDs) and cardiac resynchronisation therapy (CRT) devices often represent a lifesaving asset, but device-related infections (DRI), albeit infrequent, still represent a potential life-threatening complication [1]. Their clinical manifestation may be confined to the device pocket or to the leads or extended to the entire system and bloodstream [2].

DRI is a relevant clinical dilemma as it increases mortality (up to 35% at 5 years), morbidity and financial health care burden with an incremental cost of USD 16,500 per hospitalisation and an average total cost of USD 146,000 per CIED infection case [3,4,5].

## 2. Risk Factors for Cardiac Implantable Electronic Device Infections

The overall incidence of CIED infections ranges from 0.5% to 2.2% of patients according to different populations, type of device and time from implant [2]. CIED infection rate raise has surprisingly exceeded the heightened number of device implantations [6]. In the last 16 years, while the number of implanted electronic devices has almost doubled (95% more), the incidence of CIED infections recorded an increase of more than 200% [5]. This escalation of infections may be caused by the higher complexity of CIED recipients in terms of comorbidities and ageing (patient-related), as well as by more sophisticated techniques and longer procedural times (procedure-related) [7].

Risk factors for CIED infection have historically been classified into patient-related, procedure-related and additionally sub-classified into non-modifiable and modifiable [3]. According to actual evidence ICDs and CRT devices are more susceptible to infections than PMs (8.9 and 10 vs. 1.8 cases, respectively, per 1000 device/years) [8,9]. The number of implanted leads is critical in terms of risk of infections [10,11]. Whether this finding is related to the mere presence of additional hardware itself or reflects the complexity and duration of the procedure is unclear and still object of debates [12]. The end-stage renal disease brings the highest risk of infection among the non-modifiable patient-related factors, showing a pooled estimate odds ratio (OR) of 8.73 [13]. Other relevant risk factors from the same category are chronic corticosteroid use, history of device infection, chronic obstructive pulmonary disease (COPD), heart failure (HF), malignancy and diabetes mellitus [2]. Although the underlying mechanism is unclear, male gender seems to be associated with an increased risk, while infections in women showed a high mortality rate [14,15]. Surprisingly, there is some evidence indicating that age is inversely related to DRI [16], probably because younger patients may have a weaker immune response against low virulence organisms, and finally they go through many procedures in their lifetime [17]. Procedural time is a sizeable determinant of DRI as longer procedures are indeed independently associated with infectious complications [18]. Device replacement or upgrade, which is a non-modifiable procedure-related risk factor as well, is associated with a 2- to 5-fold risk of DRI compared with de novo implant [2]. Early re-interventions, defined as repeat procedures occurring during a single hospitalisation, are also associated with an 8.8-fold increased risk of CIED infection. Pocket hematoma, temporary pacing wires and unfamiliarity with implant techniques are all independent risk factors for DRI [19]. Modifiable factors leave room to preventive strategies to overcome the increased risk for DRI. Fever in the 24 h before implantation is certainly the most relevant (pooled estimate OR > 4). Improper trichotomy, oral anticoagulants and heparin bridging have a minor impact [3].

Ultimately, an investigation on the WRAP-IT trial population, using a machine learning analysis that considers 81 variables, identified additional non-modifiable risk factors including higher number of CIED procedures, history of atrial arrhythmia, geography (outside North America and Europe), device type (CRT vs. permanent PM/ICD) and lower body mass index. Potentially modifiable risk factors included longer procedure time, implant location (non-left pectoral subcutaneous), peri-operative glycopeptide antibiotic (vancomycin) vs. non-glycopeptide (cefazolin), anticoagulant and/or antiplatelet use and capsulectomy. Chlorhexidine skin preparation and antibiotic pocket wash have been found to be protective from early DRI [20].

## 3. Pathogenesis and Microbiology of Cardiac Implantable Electronic Device Infections

There are two key classes of clinical manifestations, device pocket infections and leads-related endocarditis. The most common source of contamination is the air or the hands of the operators and pocket infection is the prevalent expression of this pathway [3]. Direct lead seeding during bloodstream infections is the mechanism of late lead vegetation formation. The pathways for germs penetration are usually skin, mouth, gastrointestinal or urinary tract infections [21]. A retrograde progression of infection from the bloodstream to the pocket has been described, and device infection could represent the first clinical expression of a subclinical bacteraemia. It is commonly accepted, although controversial, that infections occurring within 1 year are probably due to contamination at the time of surgery, while those occurring later may be caused by blood-bared germs [7]. ICD infections generally occur earlier after the implant compared with PM infections (125 vs. 415 days, respectively) [8]. This finding is probably related to procedural time and higher susceptibility of ICD leads to shelter micro-organisms seeding. Data suggest that more than a half of DRI is related to procedural contamination. In 55% of patients indeed, a DRI is detected before 12 months after last procedure [22]. Similarly, it has been detected that 25% of CIED infections occur in the first month (0–28 days after device placement), 33% later (29 days to 1 year after device placement), and 42% of total DRI are delayed (>1 year after device placement), implying that only 4 out of 10 infections are not primarily related to peri-procedural contamination [23]. A study including only patients with lead-associated endocarditis, showed that more than two-thirds of individuals developed the disease at least 1 year after the procedure, supporting the theory that late infections are mainly lead-related and secondary to bacteraemia [24]. As expected, risk factors for DRI within 6 months of implantation are different from those related to later infections. The presence of epicardial leads or immediate peri-procedural wound complications seem to be associated with early infections, while the hospitalisation span, the presence of COPD and other comorbidities are mostly associated with delayed infections. Therefore, different pathogenetic mechanisms are associated with distinct clinical presentations, both in terms of time (early versus late) and of clinical manifestation (pocket infection versus lead/systemic infection), which in turn are burdened with different prognosis. In fact, some studies have found higher mortality in lead-related CIEDs or bloodstream infections (29%) compared with isolated pocket infection (5%) [25]. The microorganisms by far most frequently involved in DRI are Gram-positive bacteria (70–90%), especially normally non-pathogenic germs such as coagulase-negative Staphylococci (CoNS, 37.6%) that usually are skin saprophytes [26]. The second most common pathogen, namely Staphylococcus aureus (StA) (30.8%), is the most lethal. It is the most common cause of bacteraemia and early pocket infections and the one that is much prone to adhere to non-biological material creating the biofilm. The biofilm is a structured community of bacterial cells enclosed in a self-produced polymeric matrix and adherent to an inert or living surface, which prevents the effective action of host defences and the penetration of antibiotics [27]. Gram-negative bacilli and other Gram-positive cocci are rarely isolated in CIED infections. Finally, a common and critical situation, ranging from 12% to 49% of situations, is that of clinical infections with negative cultures [28].

## 4. Pocket Infections

Uncomplicated pocket infection is defined as an infection limited to the generator pocket without systemic symptoms, clinical signs of infection or positive blood cultures [2,3]. The generator pocket is the most frequent site of CIED infection [29]; indeed, almost two-thirds of patients have a localised pocket infection [8]. Whereas later stages of pocket infection are clearly evident, with discernible features, the earliest stages of the infection may be devious and easily confused with other clinical conditions, such as pocket hematoma, post-implantation inflammation or superficial wound infection. Those complications typically occur within 30 days from the index procedure and have a different therapeutic management and prognosis (Table 1). Local signs are quite different from mild inflammation (erythema, warmth, pain and swelling), in the early stage of the disease, to the real “positive clinical pocket” in later stages. Advanced pocket infection gives raise to fluctuance (abscess) and adherence of the pocket, purulent material drainage from incision sites, fistula formation, wound dehiscence and skin erosion with externalisation of the generator or leads. In this situation, the device should be considered contaminated, independently from the results of the microbiology [2]. In this setting, diagnostic percutaneous puncture with pocket fluid aspiration should be generally avoided to prevent further inoculation with bacteria [30]. Even when performed in a sterile environment, the puncture results in a skin barrier interruption creating a gateway for microbes. The clotted blood, the warm and moist setting of the pocket, enhance bacteria proliferation. Finally, pocket infections may reach the bloodstream prompting systemic infections.

**Figure 1:**
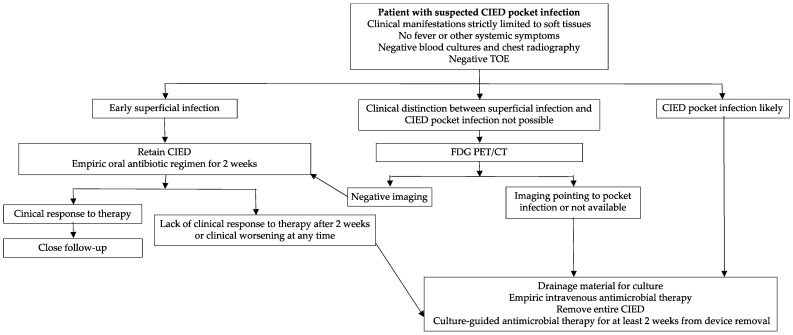
Proposed algorithm for the management of patients with suspected CIED pocket infection. CIED: cardiovascular implantable electronic device; TOE: trans-oesophageal echocardiography; FDG PET/CT: Fluorine-18-fludeoxyglucose (18F-FDG) positron emission tomography/computerised tomography.

Apparently uncomplicated pocket infections should not be assumed as localised CIED infection, as germs may follow the path along leads and cause secondary blood-stream infection and endocarditis. Current data show that when a clinical pocket infection is identified, rates of lead or endocardial involvement range between 6% and 58% [31]. Precisely, complicated generator pocket infections are internationally defined as a pocket infection with evidence of lead or endocardial involvement and/or systemic signs and/or symptoms of infection or positive blood cultures. Regardless, pocket infection is uncomplicated or complicated, current International Societies Guidelines suggest CIED removal/extraction associated with a specific antibiotic regimen (Figure 1) [2,3]. The investigation of the extent of the infection, the overall management and treatment processes traditionally overlap [17]. For these reasons, some authors consider the clinical differentiation between complicated and uncomplicated pocket infection as a mere academic exercise.

## 5. Cardiac Device-Related Infective Endocarditis and Bacteriemia

Bloodstream infection usually refers to a CDRIE which is defined as the presence of lead or valvular vegetations in combination with positive blood cultures [32]. Nevertheless, the clinical spectrum of systemic CIED infection includes two other conditions:

- A left-sided endocarditis in a CIED carrier: the therapeutic approach follows the current guidelines for valve endocarditis [33]. If surgery is required for left-sided endocarditis, an open-heart removal of the CIED is recommended regardless of the presence of acknowledged device involvement. If there is no indication for valve surgery, complete hardware extraction should be considered even if there is no evidence of associated device infection.
- An occult bacteraemia in a CIED carrier: in this case, there is not an alternative source of infection which resolves only after CIED extraction [34].

**Figure 2:**
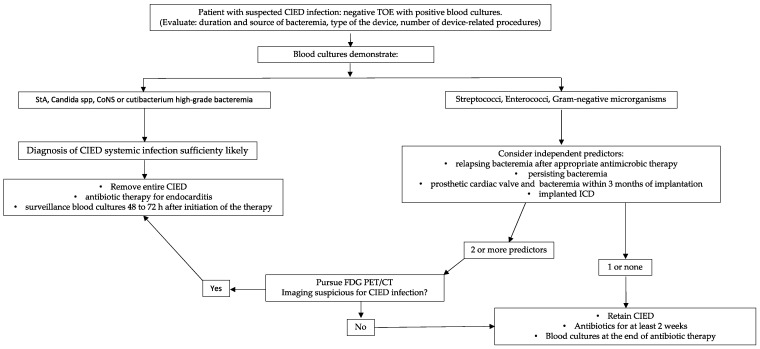
Proposed algorithm for the management of patients with suspected CIED infection with negative TOE and positive blood cultures. CIED: cardiovascular implantable electronic device; TOE: trans-oesophageal echocardiography; StA: Staphylococcus aureus; CoNS: coagulase-negative Staphylococci; ICD: implantable cardiac defibrillator; FDG PET/CT: Fluorine-18-fludeoxyglucose (18F-FDG) positron emission tomography/computerised tomography.

The diagnosis of CIED systemic infection is very challenging and it should always be suspected in case of history of fever positively responding to antibiotic therapy and relapsing after its discontinuation [35]. However, several studies have found that 20–50% of patients with CDRIE may present without systemic signs of infection, such as fever, chills, malaise or anorexia and it should spur clinicians to increase their attentiveness to CIED infections [11,23,36,37]. The most frequent complication of CDRIE is the presence of a tricuspid valve vegetation, which occurs in about one-third of the cases [38]. Tricuspid involvement can present with valve alterations (stenosis or regurgitation), pulmonary emboli or pneumonia [39,40]. Tricuspid regurgitation, which is the most prevalent, when severe may require surgical correction coupled with open-heart lead extraction. In case of small tricuspid vegetations, with mild or moderate valve insufficiency, percutaneous extraction can be performed and medical treatment continued for valve endocarditis [34]. Septic thrombophlebitis of the axillary-subclavian axis, even though it is a rare condition, can occur when multiple leads are placed through the same vessel (e.g., CRT or abandoned leads). This complication is at very high risk of pulmonary embolism and an aggressive antithrombotic therapy is recommended before the explant of the whole device [41]. The diagnosis of CDRIE is still based on the modified Duke criteria, but many studies have highlighted some criticism about their predictive value in this setting [33,42]. In order to increase the sensitivity for CIED infection diagnosis, the European Heart Rhythm Association developed the International CIED Infection Criteria in 2020; unfortunately, many limitations are still present [3]. Caution should be maintained in cases of incidental masses on leads without clinical signs of infection because they may be of thrombotic origin [43]. In this situation, four sets of blood cultures and inflammatory markers should be obtained over 2–4 days. If they are all negative, clinical and echocardiographic follow-up is warranted and anticoagulant treatment should be considered, keeping in mind that a mass on right heart CIED leads without signs of infection may also represent a malignancy [44]. Not all patients with a CIED and positive blood cultures have an underlying CIED lead infection. Individuals with positive blood cultures, but no evidence of localised CIED infection constitute a group of difficult management (Figure 2). The risk of underlying CIED lead infection in presence of bacteraemia depends on several factors including duration and source of bacteraemia, type of device, the number of device-related procedures and especially the type of microorganism isolated in blood cultures [45]. Gram-positive organisms remain the predominant pathogens associated with CIED: CoNS and StA are more prone to adhere to non-biological materials [46,47]. Moreover, StA is the most common cause of bacteraemia and early pocket infections. For this reason, many studies tried to identify the clinical predictors of underlying CDRIE in patients presenting with StA bacteraemia, but no signs of pocket infection. Uslan et al. identified different independent predictors of CIED infection such as: relapsing bacteraemia after an appropriate period of antibiotic therapy (when no other source of bacteraemia has been identified), persisting bacteraemia for more than 24 h, implanted ICD, prosthetic cardiac valve and bacteraemia within 3 months of device implantation [48].

## 6. Diagnosis of CDRIE

CDRIE is defined, according to European guidelines, as an infection extending to the electrode leads, cardiac valve leaflets or endocardial surface; however, local device infection and CDRIE are difficult to be differentiated. Clinical manifestations are the same of other forms of endocarditis, with some differences: fever is less prevalent especially in the elderly, while respiratory and rheumatological symptoms as well as local signs of infection are predominant [33]. The diagnosis should start from blood cultures, no less than two sets; three or more are recommended [49]. Suspected CIED infection with negative cultural findings should consider fungal/mycobacterial blood cultures to exclude an unrecognised causative pathogen [45]. Swab samples from the device and generator pocket tissue for culture and susceptibility testing are valuable instruments [45]. Tissue cultures acquired during the surgical exploration are more sensitive than swab cultures [49]. The use of biomarkers was investigated by Lennerz et al. concluding that pro-calcitonin and high sensitivity C-reactive protein could aid in the diagnosis [50]. Recent studies have suggested that leads and generator sonification after removal may help the microbiology testing [51].

**Figure 3:**
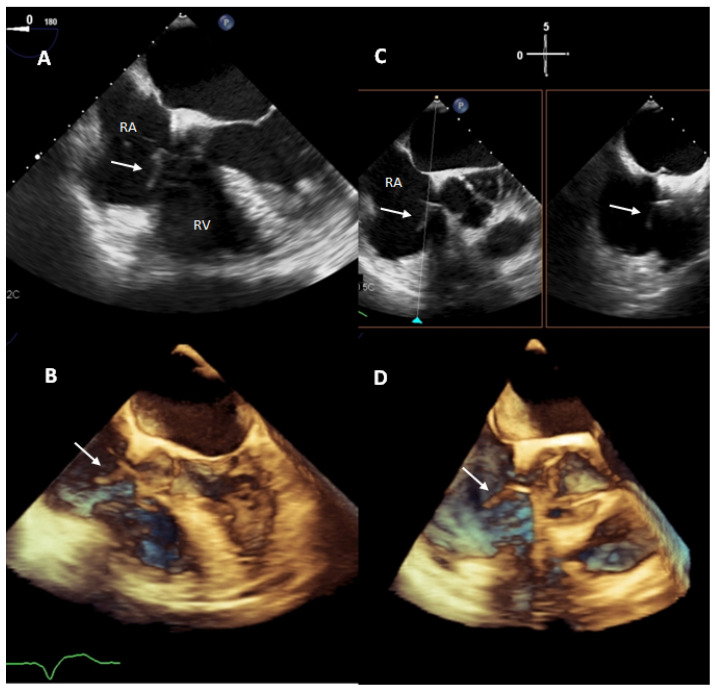
Transesophageal echocardiogram, 5 chamber view, showing a vegetation (19 × 6 mm) adherent to pacemaker’s ventricular lead in 2D (panel (**A**), white arrow) and 3D (panel (**B**)). The same vegetation is also shown in right ventricular inflow-outflow view (and its orthogonal plane-bicaval view) in 2D (panel (**C**)) and in 3D (panel (**D**)). RV: right ventricle; RA: right atrium.

Trans-thoracic (TTE) and trans-oesophageal echocardiography (TOE) are the first essential instruments of the diagnostic workup as they help sizing and follow-up of the vegetations identifying possible valvular involvement and dysfunction. TTE can detect pericardial effusion, ventricular dysfunction and pulmonary artery pressure better than TOE, while the latter would be more accurate for the diagnosis of lead-related endocarditis and peri-valvular extension of the infection (Figure 3). TTE and TOE must be performed both when CDRIE is presumed and intracardiac echocardiography may be considered in case both tests are negative [33].

When the echocardiographic investigations are negative or doubtful and the clinical suspicion is quite reasonable, Fluorine-18-fludeoxyglucose (18F-FDG) positron emission tomography/computerised tomography (PET/CT) scanning and radiolabelled leucocyte scintigraphy have been described as a complementary tool not only in the diagnosis of CDRIE, but also in the search for complications including pulmonary septic embolism [33].

**Figure 4:**
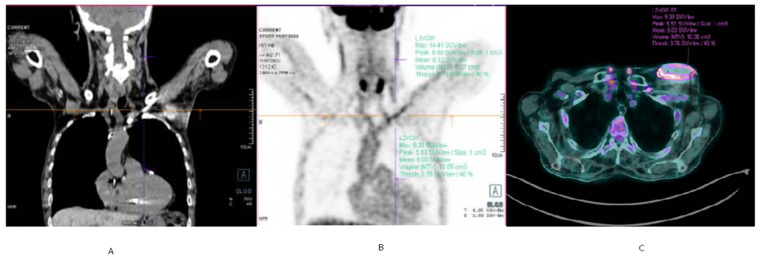
Fluorodeoxyglucose (FDG)-positron emission tomography (PET)/computed tomography (CT) of CIED pocket infection. Coronal slices of CT (panel (**A**)) and of PET (panel (**B**)) showing FDG uptake of the proximal part of the lead; fused PET/CT with abnormal FDG uptake at the pocket (panel (**C**)).

99mTc-labeled hexa-methyl-propylene-amine-oxime (HMPAO) white blood cell (WBC) scintigraphy with single-photon-emission computed tomography–computed tomography (SPECT-CT) detects and localises metabolically active cells involved in inflammation and infection. In the study by Erba et al. 99mTc-HMPAO WBC scintigraphy was 94% sensitive for both detection and localisation of CIED infection and associated complications, with a 95% negative predictive value to exclude device-associated infection during a febrile episode and sepsis [52]. 18F-FDG-PET/CT (Figure 4), conversely, performs better for pocket infections than for lead infections: for pocket infections, pooled sensitivity and specificity were 93% and 98%, respectively, while for lead infections sensitivity was 65% although specificity was still high (88%) [53]. Aside from that, delayed image acquisition could increase 18F-FDG-PET/CT diagnostic accuracy in suspected CDRIE [54]. Previous antibiotic therapy may yield false-negative PET/CT imaging despite CDRIE being present [55].

No validated clinical tools are available to date, EHRA has proposed to combine modified Duke and ESC 2015 criteria [33]. Positive lead-culture is the major criterion to establish CDRIE among those proposed in the modified Duke [36]. Nevertheless, the use of positive lead cultures can be misleading as the lead tip could be contaminated during the extraction passing through the infected pocket. In addition, previous antibiotics administration and biofilm protection could affect culture sensitivity preventing colony formation [56].

## 7. Device Removal Versus Device Retention

Key aspects in the management of CIED infections are rapid diagnosis and timely treatment. Early intervention, performed within three days of diagnosis, conducts to a significant reduction in in-hospital mortality [57]. A large-population cohort study reported high short- and medium-term complication rates related to CIED removal. However, at the multivariate analysis, the use of initial antimicrobial therapy as unique strategy was associated with a 7-fold increase in 30-day mortality, while immediate device removal showed a 3-fold decrease in 1-year mortality compared to delayed extraction [58].

International position and consensus documents highlight how effective treatments require complete removal of the system and any transvenous or subcutaneous component as well as any residual non-functional lead. A significant cause of relapses is represented, in fact, by retained hardware.

Complete device extraction should be performed when a valve is replaced or repaired for infective endocarditis since CIEDs could serve as a pabulum for pathogen’s persistence and multiplication [59].

Lead vegetation recorded through TOE, after having ruled out the presence of a non-infected fibrin stranding (a very common finding in long-duration leads) is an absolute indication for device extraction [3].

Device removal outcomes for CDRIE does not differ in elderly and younger population [60]. The laser excimers devices appear to be more incline to vascular damage particularly involving the superior vena cava [61].

The therapeutic strategy to be adopted in presence of positive blood cultures varies depending on the microorganism that is found. A single positive test for StA (especially within three months of CIED manipulation or in case of recurrences despite specific antimicrobial therapy) or Candida species is enough to suggest the extraction of the system. On the other hand, in presence of CoNS, Cutibacterium or other pathogens commonly causing endocarditis, high-grade bacteraemia (two or more separate blood cultures positive for the same organism) is required to have a specific diagnosis. A single positive blood culture for one of the last listed pathogens may represent skin contamination. However, if the clinical suspicion persists, it may be reasonable to perform other diagnostic imaging tests such as 18F-FDG PET/CT and to discuss the subsequent management with an infectious disease expert [3,62]. In the aforementioned cases, the procedural risks related to CIED removal are significantly lower than the rate of mortality or recurrence of infection even if alternative strategies are adopted, such as antibiotic therapy or generator extraction with retention of the leads [63].

Signs and symptoms of pocket infection including oedema, erythema, purulent drainage, skin erosion with exposure of the generator or leads and pain, are a warning of the need to remove the device, even in absence of a positive culture of the drainage of the wound or bacteriemia [64]. Superficial incision infections, especially if they occur in the first weeks after implantation, with the involvement of the skin and the subcutaneous tissue without the participation of the fascia and the muscle, provide for close monitoring for one to two weeks to rule out the progression to deeper tissues which would require extraction. A similar approach should be adopted in presence of pocket hematoma. Some authors suggest the opportunity to start an empiric antibiotic therapy with activity against staphylococcus spp. [49].

Where indicated, transvenous removal of all leads, including the abandoned ones, is the most recommended technique with low rates of mortality and major complications [2]. Usually, leads implanted at least two years earlier are technically more difficult to extract and the procedure should be performed by experienced operators. Different types of leads involve different challenges during the removal: ICD leads, with the presence of one or two coils steering to the formation of more extensive adherences, are more prone to procedural complication, especially when a caval coil is present; the same applies to those with passive fixing compared to those with active fixing.

The evaluation of the balance between the surgical risk of removal and the benefits in terms of eradication of the infection is mandatory in presence of epicardial leads or patches connected to pectoral or abdominal generators. When the contamination is isolated to the pocket, an option is to perform a separate incision away from the pocket, adjacent to the thoracic entrance of the epicardial leads or patches, and to cut their connection with the generator. Their proximal end can then be removed from the pocket [65].

Large vegetations should be carefully assessed in terms of risk for pulmonary embolism with the transvenous method and the risk-benefit balance of a surgical procedure. The surgical threshold, in fact, has still to be defined and this approach is associated with greater morbidity [57]. Observational studies with small sample sizes, on the other hand, showed low rates of hemodynamically relevant pulmonary embolism with transvenous removal independently from vegetative mass dimensions [66,67]. Percutaneous removal of such vegetations, with a suction and debulking technique, before lead transvenous extraction, has been reported [68]. Filtering of vegetations using an in-line filtered veno-venous extracorporeal circulation has been described for very large masses. This technique shows beneficial effects reducing post-operative sepsis or pneumonia related to small vegetations embolisation [69].

Pocket management is likewise a crucial aspect of device removal. It requires an accurate debridement with complete excision of the fibrotic capsule and of the non-adsorbable suture material and following plenty sterile saline irrigation [70].

Sometimes the removal of the CIED is desirable, but not feasible. It is the case of patients with relevant comorbidities, limited life-expectancy, with devices implanted for a long time and with PM dependency. Evidence is limited in this setting. Current recommendations are that such individuals should undergo full targeted antibiotic therapy for at least six weeks as last resort. In most cases they experience limited survival rates and high likelihood of relapses [71].

CIED retention strategy is limited to few instances where the infection of the system is unlikely. For example, when the established pathogen in the blood stream is not a StA, the definition of CIED or valvular vegetation matters. With this kind of setting, imaging techniques should identify the vegetations location, pocket infection should be excluded, and the device should not have been manipulated for less than three months.

CIED retention may also be considered in case of skin incision site infections with superficial cellulitis or stitch abscess, without the involvement of the pocket. Such a diagnosis is often difficult, and these patients should be carefully followed up. The surgical technique used for superficial infection should include wide excision of healthy skin around the infected area and must be performed in a proper sterile environment. The first manoeuvre, crucial in the analysis of the infected wound, must be the verification of a possible communication between the pocket of the device and the outside. If the integrity of the pocket is compromised, total removal of the system deserves to be considered [49].

**Figure 5:**
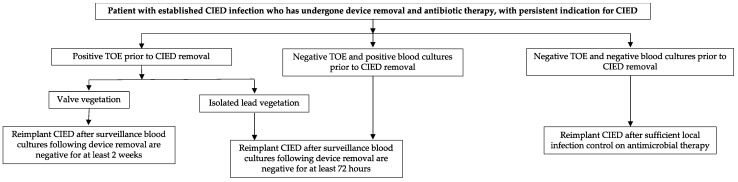
Proposed algorithm for optimal re-implantation timing in patients with persistent indication to CIED. CIED: cardiovascular implantable electronic device; TOE: trans-oesophageal echocardiography.

The definition of optimal re-implantation timing requires further analysis (Figure 5). About one third of patients who undergo CIED extraction do not require a new device. A temporary system, when the need to place a cardiac pacing device is not deferrable, could be implanted, considering that this increases the infectious risk. Individuals who showed valve vegetations on TOE should be implanted after at least 14 days from last negative blood cultures. In case of isolated lead involvement or in case of bacteraemia without demonstration of vegetations on TOE, waiting 72 h from last negative blood cultures could be enough [3]. Finally, as soon as an isolated pocket infection has completely healed, new CIED positioning is possible. In the case of PM-dependent patients, local pocket infection may be managed with device extraction and, once specific antibiotic regimen has been started, contralateral device implant may be considered.

Different devices as subcutaneous ICDs (S-ICDs) or leadless PMs may also be taken into account to reduce the risk of relapses [49].

Due to their recent introduction into clinical practice, little data is available on the risk of infection for leadless PMs. However, when compared with conventional permanent pacing, they showed lower rates and a lower predisposition to hematogenous infections even when implanted in the presence of active bacteraemia [72,73]. The reason could be related to the specific coating system of the devices, the small surface area and the progressive fibrous encapsulation in the right ventricle which make them more resistant to bacterial seeding. Furthermore, the absence of the generator pocket and the use of a delivery system avoids physical handling of the device during implantation [73].

## 8. Antimicrobial Therapy

Although randomised studies are currently lacking, resolutive treatment of CIED infections requires early and complementary association between complete system removal and antimicrobial therapy [2,33,49,74].

Multiple antimicrobial regimens may be considered, depending on several different clinical scenarios. The available data are mostly based on in vitro susceptibility, observational studies, pharmacokinetic/pharmacodynamic data and clinical experience. Antimicrobial choice for CIED infection depends on multiple factors such as severity of clinical presentation, plans for device management, cardiac involvement, extra-cardiac foci of infection, allergy, concurrent medications and renal impairment. An appropriate treatment should therefore be discussed by a multidisciplinary team with expertise in CIED infection (i.e., “Endocarditis Team”) [2].

Even though empirical treatments (before the pathogen identification) cover a broad spectrum of bacilli, sometimes requiring complex and potentially toxic antimicrobial associations, they are usually less effective than “targeted” approaches. Potential life-threatening conditions as severe sepsis/septic shock require an empirical treatment urgently after sampling for blood cultures to minimise the prognostic impact of systemic involvement. On the other hand, many CIED-related infections show indolent clinical course enabling, whenever possible, to await cultures report and susceptibility testing to set up a “targeted” strategy. Antimicrobial regimens should be kept as simple as possible until microbiological results and CIED system management are defined. Antibiotic treatment recommendations are summarised in (Table 2), as listed in the 2020 EHRA international consensus document [2].

Successful antibiotic rescue therapy and long-term suppressive antibiotic therapy have been used in selected cases when device removal is considered contraindicated, but there is only limited clinical experience reinforcing the need of multidisciplinary “Endocarditis Team” management [71,74].

Finally, yeasts are a rare cause of CIED infections and routine empirical antifungal therapy is generally not recommended. Candida species are the most frequently involved (ranging from C. albicans to C. parapsilosis). When clinical features suggest fungal aetiology and require urgent treatment initiation, empirical approach should include amphotericin B with/without 5-flucytosine or an echinocandin agent such as micafungin as primary therapy. In stable patients with documented susceptible microorganism and negative blood cultures, step-down therapy with fluconazole 400–800 mg daily could be a reasonable choice [75].

Administration of antimicrobial treatment should be managed orally or intravenously according to patients’ clinical features and needs. Although no clinical trial is available on this topic, there is a wide consensus advising that IV therapy is the best choice for CDRIE and attempted CIED salvage, while oral treatment could be more appropriate in localised pocket infections and after system removal. Central venous catheterisation is preferably to be avoided, except when requested for clinical instability or difficult peripheral access. The peripheral venous cannula has the lowest infection potential (with 72h replacement) and reduces the risk of damaging future sites for CIED implantation. When long-term IV therapy is planned, peripherally inserted central catheter (PICC) or ‘midline’ (MID) is preferable [3]. Vascular accesses-related risk of infections shows a direct correlation with the permanence of the cannula, and periodic switches between peripheral cannula and PICC/MID should be planned by nursing professionals in order to manage this risk [2].

## 9. Prevention of CDRIE

Even if there are no large, controlled studies on this topic, antibiotic prophylaxis is recommended before implantation of CIEDs. A first-generation cephalosporin, such as cefazolin, is usually used as prophylaxis and should be parenterally administered one hour before the procedure. Vancomycin, teicoplanin and daptomycin may be considered instead of cefazolin in centres where oxacillin resistance among staphylococci is high, in high-risk patients or in patients with contraindications to cephalosporins. They should be started before the procedure according to the drug pharmacokinetics [33]. In patients who are allergic to both cephalosporins and vancomycin, daptomycin and linezolid represent an option [49]. Potential sources of sepsis should be eliminated at least two weeks before implantation in deferrable procedures [33].

Antibiotic prophylaxis before invasive procedures that are not directly related to CIEDs manipulation is not recommended based on limited evidence. Furthermore, the predominant pathogens in CDRIE are staphylococci, which are different from the expected pathogen associated with translocation during dental, gastrointestinal, or genitourinary procedures. Post-procedural antibiotics are not recommended given the lack of evidence in terms of benefits and potential risks.

According to the PADIT trial, incremental administration of antibiotics on the basis of the clinical risk of CDRIE does not significantly impact the prevalence of infections [76].

An interesting role could be played by antimicrobial eluting antibacterial envelopes. The TYRX^TM^ (MEDTRONIC TYRX^TM^ Inc. New Jersey USA) antibacterial envelope (the last available type) is a monofilament polypropylene mesh that holds the cardiac implantable electronic device in place and emits rifampin and minocycline slowly over time [51]. More recently, two prospective cohort studies were conducted to evaluate the use of antibacterial envelope among high-risk patients receiving ICD and CRT. These studies showed a very low infection rate of 0.4%, which was significantly lower than the 12-month benchmark rate of 2.2% [77]. Moreover, a large randomised clinical trial enrolling patients from multiple sites across the world reported a 40% reduction in major CDRIE within 12 months after the procedure with the use of antibacterial envelopes [78].

Pocket hematoma that complicates CIEDs placement or invasive manipulation has been identified as a risk factor for infection [79]. Prevention of hematoma during the procedure is desirable: meticulous cautery of bleeding sites, application of topical thrombin, irrigation of the pocket, the use of monofilament suture for sub-cuticular layer, a pressure dressing applied for 12 to 24 h after skin closure may decrease the risk of hematoma formation [49]. The “bridging approach” with anticoagulation increases the risk of hematoma and should be avoided in CIEDs-related procedures [80]. For patients undergoing device implantation, prospective and randomised data in vitamin K antagonists (VKA)-treated patients indicated lower thromboembolic and bleeding rates if the VKA is continued, without any bridging [81]. For Direct Oral Anticoagulant (DOAC)- treated patients, the BRUISE-CONTROL 2 trial demonstrated similar bleeding and embolic rates in patients with a last intake two days before the implantation compared to those who continued DOAC until the morning of the procedure [82]. A balance between thrombotic and bleeding risks must be pursued: to stop DOAC the day before the procedure seems reasonable in most cases, especially when bleeding risk exceeds stroke risk. Resumption of DOAC regimen on the first postoperative day is usually feasible [83].

Patients on dual antiplatelet therapy carry an increased risk of post-operative pocket hematoma compared to patients treated with aspirin alone or without antiplatelet therapy. In such cases, European guidelines recommend P2Y12 receptor-inhibitors discontinuation for 3–7 days (according to the specific drug) before the procedure, where possible, accordingly to an individualised risk assessment [80].

Needle aspiration should be avoided because of the risk of introducing skin flora into the pocket with the subsequent development of infection [49].

In elective procedures, StA colonisation can be detected by nasal swabs. Nasal treatment with mupirocin and chlorhexidine skin washing reduce colonisation and should be preferred to iodine solution [20,76].

AHA/ACC/HRS guidelines suggest a S-ICD in patients who are at high risk for infection and in whom pacing is neither needed nor anticipated [84]. Even if the CIED infection rate for S-ICDs is still not demonstrated to be lower than transvenous ICDs, the absence of the possibility for infective endocarditis with S-ICDs is the reason for this recommendation [85]. When available and according to the clinical indication, leadless PMs carry a lower incidence of CDRIE [86].

A summary of possible strategies to prevent CDRIE is available in Table 3.

## 10. Conclusions

Infections represent one of the main factors of mortality and morbidity that afflict patients with CIEDs. Their correct definition and an appropriate diagnosis allow a precise treatment in terms of removal or retention of the device, antimicrobial therapy and optimal timing of re-implantation. Nowadays, however, there are alternative strategies and prevention mechanisms that must be employed and implemented to reduce the burden of this problem.

## Figures and Tables

**Table 1:** Differential diagnosis among cardiac implantable electronic device pocket complications.

Clinical Entity	Characteristics	Incidence	Time Period after Implant	Prognosis (+ Good Prognosis, − Bad Prognosis)	Management
Pocket Hematoma	Ecchymosis, mild effusion in the pocket and swelling.	1–20%	Within 2 weeks (usually <48 h)	+	Compression bandage, removal of antithrombotic therapy, specific pocket compression vest.
Post-implantation inflammation	Erythema affecting the incision site, without purulent exudate, dehiscence, fluctuance or systemic signs of infection.	1–10%	Within 30 days (usually <7 days)	+ +	Close observation. Antibiotics not mandatory.
Superficial infection of surgical wound	A small, localised area of erythema and/or purulence associated with a suture defect.	0.5–5%	Within 30 days (usually <14 days)	+/−	Removal of the suture and antimicrobial therapy, if indicated.
Uncomplicated pocket infection	From mild inflammation to deformation, fluctuance, adherence of pocket, purulent material drainage from incision sites, fistula formation, wound dehiscence and exposure of the generator or leads.	0.5–2.2%	Whenever, traditionally within 1 year	−	Cardiac implantable electronic device removal/extraction associated with a specific antibiotic regimen.

**Table 2:** Antibiotic treatment recommendation.

**Surgical Incision Infection**	
**Empirical Treatment**	
Oral antibiotic covering StA:	flucloxacillin 1 gr (every 6–8 h)
If high MRSA prevalence:	trimethoprim-sulfamethoxazole, clyndamicin, doxyciclin, linezolid
Targeted after culture results	
Duration: 7–10 days	
**Isolated Pocket Infection**	
**Empirical Treatment**	
Intravenous treatment covering StA and multi-resistant CoNS	vancomycin 30–60 mg/kg/day i.v. in 2–3 doses daptomycin 8–10 mg/kg i.v. o.d.)
If systemic symptoms	
For additional Gram-negative coverage, combine with 3rd generation cephalosporin (or broader beta-lactam) or gentamicin	cephalosporins standard dose or gentamicin 5–7 mg/kg i.v. o.d.
Targeted after culture results	
If sensitive Staphylococci:	flucloxacillin 8 g/day i.v. in 4 doses or 1st generation cephalosporins (standard dose)
Targeted after culture results	
Duration post-extraction: 10–14 days	
**Systemic Infections**	
**Without Vegetation on Leads or Valves ± Pocket Infection**	
Empirical combination treatment covering multi-resistant Staphylococci and Gram-negative bacteria	vancomycin 30–60 mg/kg/day i.v. in 2–3 doses (alternative: daptomicin 8–10 mg/kg i.v. o.d.) plus 3rd generation cephalosporins standard dose i.v (or broader beta-lactam) or gentamicin 5–7 mg/kg i.v. o.d.
Targeted after culture results	
If sensitive Staphylococci	flucloxacillin 8 g/day i.v. in 4 doses 1st generation cephalosporin (standard dose)
Targeted after culture results	
Duration post-extraction: 4 weeks (2 weeks if negative blood cultures)	
**CIED endocarditis with vegetation on leads and/or valves ± embolism**	
Empirical treatment	vancomycin 30–60 mg/kg/day i.v. in 2–3 doses (alternative: daptomicin 8–10 mg/kg i.v. o.d.) plus 3rd generation cephalosporins standard dose i.v (or broader beta-lactam) or gentamicin 5–7 mg/kg i.v. o.d.
If prosthetic valve and staphylococcal infection	add rifampicin after 5–7 days, 900–1200 mg/day in two doses (orally or i.v.)
Adjust to culture results according to ESC Endocarditis Guidelines	
Duration: - for native valve infective endocarditis: 4 weeks post-extraction - for prosthetic valve endocarditis: 4 to 6 weeks - for isolated lead vegetation: 2 weeks after extraction may be sufficient (4 weeks in total) except for StA infection	
**Bacteraemia in a CIED patient without signs of pocket infection or echocardiographic evidence of lead or valve involvement**	
According to pathogen-specific treatment guidelines	
**Attempted Salvage Therapy and Long-Term Suppressive Therapy**	
I.v. antibiotics as in prosthetic valve endocarditis for 4–6 weeks	
Stop antibiotic therapy under close follow-up or continue individualised long-term suppressive oral therapy	

Adapted from Blomstrom-Lundqvist C. et al. European Heart Journal (2020) 41, 2012–2032. CIED: cardiovascular implantable electronic device; StA: Staphylococcus aureus; MRSA: methicillin-resistant Staphylococcus aureus; CoNS: coagulase-negative Staphylococci; i.v.: intravenous; o.d.: once daily.

**Table 3:** Preventive strategies for cardiac device-related infective endocarditis.

Strategy	Description
**Antibiotic prophylaxis before CIED implantation**	A first-generation cephalosporin. Vancomycin, teicoplanin and daptomicin in patients with contraindications to cephalosporins.
**Antibiotic prophylaxis before other procedures**	It is not recommended based on limited evidence
**Antibacterial envelope**	Reduces the rate of CDRIE
**Hematoma pocket prevention**	It is a risk factor for infection. Needle aspiration should be avoided.
**Nasal swab**	No studies for patients with CIED

CIED: cardiovascular implantable electronic device; CDRIE: cardiac device-related infective endocarditis.

## Data Availability

Not applicable.
